# Tracing the failing heart: dual genetic fate mapping for target identification

**DOI:** 10.1038/s41392-023-01564-8

**Published:** 2023-08-04

**Authors:** Yaw Asare, Christian Stoppe, Jürgen Bernhagen

**Affiliations:** 1 Institute for Stroke and Dementia Research (ISD), Klinikum der Universität München (KUM), Ludwig-Maximilian-University (LMU), 81377 Munich, Germany; 2 Department of Anesthesiology, Intensive Care, Emergency and Pain Medicine, University Hospital Würzburg, 97080 Würzburg, Germany; 3 Department of Cardiac Anesthesiology and Intensive Care Medicine, German Heart Center Charité Berlin, 13353 Berlin, Germany; 4 Munich Cluster for Systems Neurology, 81377 Munich, Germany; 5 Munich Heart Alliance, 80802 Munich, Germany

**Keywords:** Cardiology, Genetics research

In a recent study published in *Nature Genetics*,^1^ Han and colleagues employed a dual genetic lineage tracing approach in combination with a model of heart failure (HF) to identify a key sub-population of endocardium-derived fibroblasts, which gives rise to excessive myofibroblast formation in a Wnt pathway-mediated manner and contributes to cardiac fibrosis.^1^ The identified fibroblast sub-population and Wnt signaling pathway could be novel targets for the therapy of HF.

**Fig. 1:**
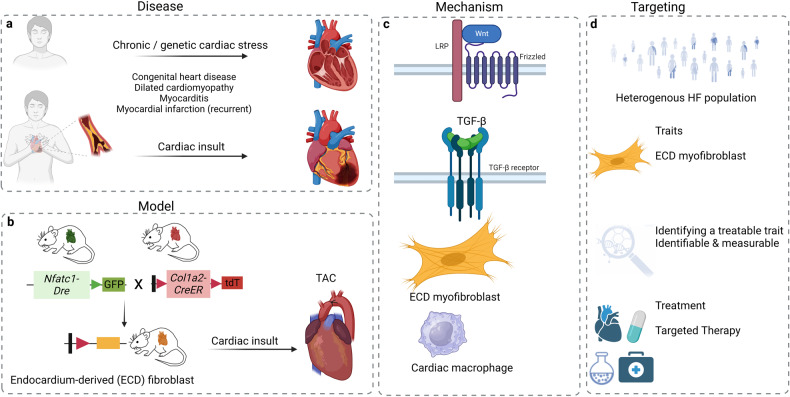
Genetic tracing and molecular mechanisms in heart failure. **a** Chronic/genetic cardiac stress or acute cardiac insults are key triggers of heart failure (HF). **b** Genetic fate mapping has emerged as a powerful resource for tracing specific cell populations in the developing heart and in models of cardiac pressure overload as exemplified here by dual genetic tracing of endocardium-derived fibroblast. **c** Key pathways implicated in cardiac development and fibrosis include the canonical Wnt signaling, and TGF-β cascade. During cardiac remodeling in HF, the Wnt signaling pathway promotes activation and expansion of endocardium-derived fibroblasts while transient activation of the TGF-β cascade drives ECM-preserving phenotypes in fibroblasts. Both cardiac fibroblasts and macrophages play important roles in the response of the heart to injury and stress, but chronic activation can lead to HF. **d**, Identifying disease-specific cell sub-types, studying the interaction of cardiac cells with each other and other cell types will help in understanding the complexity of the repair mechanism following cardiac injury and guide strategies to identify and target treatable traits, with valuable diagnostic and therapeutic implications. Abbreviations: ECD, endocardium-derived; TAC, transverse aortic constriction; TGF-β, transforming growth factor-beta; Nfatc1, nuclear factor of activated T cells-1; Dre, D6 site-specific DNA recombinase; Col1a2, collagen type I alpha 2 chain; tdT, tandem dimer tomato. *Created with BioRender.com*

Improvements in risk management and cardiovascular intervention have significantly reduced age-specific cardiovascular disease-related mortality. In contrast, the rate of HF-related hospitalisations has increased over the past decades and HF is a disease with highest social and economic cost in industrialized countries. HF is defined as impairment of the heart’s blood pumping function and its severity is assessed based on ejection fraction. HF often occurs after myocardial infarction (MI), with infarct size and quality of ventricular remodeling after MI influencing ventricular dysfunctions. Causes of HF also include hypertension, atrial fibrillation, and cardiomyopathies (Fig. 1). Especially elderly patients with recurrent MI and comorbidities are increasingly presenting with signs of HF.

Post-infarct healing and ventricular remodeling are crucial to preserve cardiac function and determine the development of HF. However, the underlying factors are largely unknown and there exists a need to improve our mechanistic understanding to ultimately improve the mid-to-long-term outcomes of HF patients. It is important: i) to identify specific phenotypes of vulnerable patients, who benefit from specific interventions; ii) to identify strategies to limit aggravation of cardiac damage by targeting specific cell populations; iii) and for MI-related HF, to further optimize infarct healing to prevent adverse remodeling.

In their recent article,^1^ Han and colleagues used genetic lineage tracing to label and track endocardium-derived fibroblasts (EDFs) in the heart. They generated mice with a dual tamoxifen-inducible genetic tracing-system (*Nfatc1-Dre; Col1a2-CreER:R26-RL-GFP*) to target endocardium-derived cells and fibroblasts (Fig. 1). The experiments revealed that fibroblast fate was determined early in development followed by expansion. They further observed a regional spread of EDFs in the valves and limited other regions of the myocardium. Cardiac fibroblasts maintain the myocardial extracellular matrix (ECM) in a healthy state, securing heart function by producing a properly structured ECM. However, initially important during repair, e.g. after MI or pressure overload, overactive cardiac fibroblasts produce excessive amounts of ECM, leading to fibrosis, characterized by excessive scarring and reduced heart function and HF.^2^

To study cardiac fibrosis, authors performed a mouse model of transverse aortic constriction (TAC) to mimic pressure overload in the heart. To determine the role of EDFs, they generated a sophisticated dual color-labeled model (*Nfatc1-Dre;Col1a2-CreER;R26-RL-GFP;R26-LSL-tdTomato*) to discern endocardium- (GFP + ) from epicardium (tdTomato + )-derived fibroblasts (Fig. 1). They found that GFP+ EDFs were enriched in the ventricular septum and inner left ventricle wall, mirroring the fibrosis pattern. They also observed significant expansion of EDFs after pressure overload and, using a Ki67 + genetic tracer, found that these fibroblasts had a higher proliferation rate compared to GFP- fibroblasts.

The authors further investigated the role of EDFs in cardiac fibrosis by generating fibroblast-diphtheria-toxin receptor (DTR) mice (*Nfatc1-Dre;Col1a2-CreER;R26-LSL-RSR-tdT-DTR*), verifying DTR-mediated ablation of endocardium-derived fibroblasts by genetic tdT color-labeling. Importantly, Sirius red staining revealed genetic deletion of these cells reduced the extent of cardiac fibrosis after TAC and improved heart function.

To explore the mechanisms involved in EDF expansion during cardiac fibrosis, the authors prepared GFP + and GFP- fibroblasts and compared their gene expression signature. They found positive regulation of the canonical Wnt signaling pathway and proliferation in GFP + fibroblasts, suggesting the Wnt pathway as a potential driver. To study its causal role in fibroblast expansion, *Ctnnb1*, the gene encoding β-catenin and the key component of the Wnt pathway, was deleted in EDFs. This led to lower β-catenin protein levels in GFP + fibroblasts. Following TAC, Sirius red staining indicated less myocardial fibrosis in *Ctnnb1* mutant compared to control mice. The Wnt pathway is best known for its role in morphogenesis and embryogenesis and has been found to become activated in organ injury and regeneration. However, its specific role in cardiac fibrosis is incompletely understood. The study by Han and colleagues now provides compelling evidence that Wnt signaling plays an important role in the expansion of EDFs during cardiac fibrosis and directly links it to HF.

The TGF-β cascade is another primary pathway involved in cardiac fibroblast activation and differentiation. Transient activation of TGF-β favorably affects the repair of the infarcted heart through its anti-inflammatory and pro-fibrogenic properties. In contrast, prolonged activation exacerbates adverse remodeling and cardiac dysfunction (Fig. 1). In response to pressure overload, the TGF-β pathway is activated with an initial protective effect by promoting an ECM-preserving phenotype in fibroblasts. Persistent signaling in cardiomyocytes and fibroblasts may lead to fibrosis and cardiac dysfunction.^3^ In addition to the TGF-β and Wnt/β-catenin pathways, the MAPK pathway, mitochondrial and metabolic signals, or the complement system have been implicated in cardiac fibroblast activation, differentiation, and dysregulation.^4^ Hence, a network of pathways likely controls cardiac fibrosis^4^ and understanding their precise interplay may facilitate the development of diagnostic and therapeutic strategies for the treatment of cardiac fibrosis.^2^

Although the study by Han et al. is limited to the pathogenesis of cardiac fibrosis following pressure overload, as mimicked in the TAC model, it provides compelling insights into the role of EDF in cardiac fibrosis. However, the restricted regional distribution of these cells also implies that non-endocardium-derived fibroblasts may contribute to cardiac fibrosis in other regions of the adult heart. Still, the data by Han et al. overall suggest that targeting endocardium-derived fibroblasts and/or Wnt signaling in this fibroblast-subtype may have diagnostic and therapeutic implications in HF. It is likely that the conclusions are translatable to other models of HF such as MI-triggered adverse ventricular remodeling. Indeed, reactivation of Wnt signaling upon ischemic injury is well described. The work by Han and colleagues also highlights the importance of complex genetic tracing strategies in understanding cellular fate and function in complex biological systems.

Heart failure is a syndrome related to an impairment of the heart’s ventricular ejection fraction. The underlying cause is often multifactorial^2,4^ and a variety of markers/modalities are employed for diagnostics.^4^ To improve diagnostic precision in HF patients, ‘the concept of treatable trait, a certain physiologic derangement characterized by biomarkers or biological changes that portend a predictable response to a therapy’ has been proposed.^5^ Although biomarkers often refer to defined laboratory tests, the term is used more broadly. It is important that the trait is measurable and corresponds with the modifiable insult or physiological process, which causes the adverse effect and is linked to a treatment response.^5^ As such, and despite the limitations of disease-mimicking mouse models, the cell type of endocardium-derived myofibroblasts identified by Han and colleagues in conjunction with the Wnt pathway driving the expansion of these pro-fibrotic cells could be promising diagnostic and therapeutic targets and may in the future qualify as treatable traits for adverse cardiac remodeling and HF.
